# The validity and reliability of the Chinese version of the biological rhythms interview of assessment in neuropsychiatry in the community: a large Chinese college student population

**DOI:** 10.3389/fpsyt.2024.1344850

**Published:** 2024-05-13

**Authors:** Hebin Huang, Xinhe Tian, Bess Yin-Hung Lam, Weicong Lu, Xiaoyue Li, Shuixiu He, Xingjian Xu, Ruoxi Zhang, Runhua Wang, Danpin Li, Yanling Gao, Ningning Chen, Shiyun Wu, Guiyun Xu, Kangguang Lin

**Affiliations:** ^1^ Department of Affective Disorder, The Affiliated Brain Hospital of Guangzhou Medical University, Guangzhou, Guangdong, China; ^2^ Department of Counselling and Psychology, Hong Kong Shue Yan University, Hong Kong, Hong Kong SAR, China; ^3^ School of Health and Life Sciences, University of Health and Rehabilitation Sciences, Shinan district, Qingdao, Shandong, China; ^4^ Key Laboratory of Neurogenetics and Channelopathies of Guangdong Province and the Ministry of Education of China, Guangzhou Medical University, Guangzhou, China

**Keywords:** depressive symptoms, biological rhythm, self-report questionnaire, validation, C-BRIAN

## Abstract

**Objective:**

To test the psychometric properties of the Chinese version of the biological rhythms interview of assessment in neuropsychiatry (C-BRIAN) in a group of young adults with and without depressive symptoms.

**Methods:**

Three hundred and seventy-eight university students were recruited as participants. Based on the scores from Center for Epidemiological Survey Depression Scale (CES-D), students were divided into the depressed group and healthy group. Explorative factor analysis was applied to assess the construct validity of the C-BRIAN. The Pittsburgh Sleep Quality Index (PSQI) and CES-D were compared with the C-BRIAN to test the convergent validity. The internal consistency of the C-BRIAN was also examined.

**Results:**

Three factors were extracted (activities, eating patterns, and sleep factors) explaining 63.9% of the total variance. The internal consistencies were very good with a coefficient of 0.94 (overall) and 0.89–0.91 for three factors. The domains of activities, eating patterns, and sleep were moderately correlated with PSQI (*r*=0.579) and CES-D (*r*=0.559) (ps<0.01).

**Conclusion:**

Our findings suggest that C-BRIAN has good validity and reliability which can be used to assess the biological rhythm in the young adult population with depressive symptoms. C-BRIAN would be a reliable tool to detect depressive symptoms for timely prevention and intervention in the community.

## Introduction

The global rate of depression among university students is approximately 28.4–30.6% (1, 2) and it was about 31–33% in the Chinese sample (3). However, it was much higher after the outbreak of the COVID-19 pandemic. For instance, it was found that 48.4% of Chinese university students in Hong Kong reached the “at-risk” threshold for clinical depression (4). Depression is a common problem among university students, but the nature of depression is multi-faceted. Prior studies showed that rhythm disturbances may be involved in the development of depression (5–8). These are endogenous daily cycles of physiological processes and behavior regulated by the oscillation of the molecular clock in the brain. The shifted phase of circadian genes’ expression and attenuated interaction between them has been found in depressive patients, indicating the potentially important role of rhythm in the pathophysiology (9). These rhythm disturbances could manifest prior to the onset of mood symptoms or disorders (10). Moreover, the levels of rhythm disturbances might not only deteriorate the severity of mood symptoms (11, 12), but also can lead to a relapse of depression (13). Therefore, there is an emerging need for the development of an assessment tool with good validity construct to measure biological rhythms which would help detect depressive symptoms for timely prevention and intervention.

Previous investigation of the relationship between circadian rhythms and mood symptoms in animal and human studies helps enhance the understanding of the causal link between circadian clock and mood disorders, which gives insight to mood disorder treatment. For instance, Imamura and Takumi (8) suggested that disrupted circadian rhythms led to depression-related responses in rodents. Along the same line, Bauducco et al. (14) found that the misalignment between circadian timing and preference might pose a risk for depression. Moreover, sleep disorders and changes in biological rhythms were suggested to have a bidirectional relationship with depression (15). These findings indicate that targeting the circadian rhythms might provide promising results in treating mood symptoms such as depression and bipolar disorders.

Previously, several assessment tools have been used to assess specific aspects of circadian rhythms, such as the Pittsburgh Sleep Quality Index (PSQI) evaluating sleep disturbance (16) and the Morningness-Eveningness Questionnaire (MEQ) identifying chronotype (17). However, both PSQI and MEQ measured a part of biological rhythms while a broader perspective of the rhythms was lacking. More recently, the Biological Rhythms Interview of Assessment in Neuropsychiatry (BRIAN) tapping on a more comprehensive construct has been developed and validated to evaluate biological rhythms in clinical settings for patients with bipolar disorder (18) and depression (19). Specifically, previous study showed that the BRIAN scores were negatively associated with one’s level of nocturnal melatonin – a biomarker that reflects circadian rhythm – in patients with major depressive disorder and bipolar disorder, suggesting promising external validity (19). Moreover, the Japanese and Korean versions of the BRIAN have been validated as having good psychometric properties (20, 21). In Chinese patients with Major Depressive Disorder (MDD) aged 40 years, the validity was also established (22). However, the validity and reliability pertaining to the Chinese version of BRIAN in college student population having depressive symptoms has not yet been established. Further investigation of C-BRIAN is warranted so to help the detection of depressive symptoms for timely and efficient treatment in Chinese college individuals who are vulnerable for depressive symptoms.

Given the rise of the need to develop a reliable and valid assessment tool for the comprehensive biological rhythms in the general public particularly young adults, the aim of the present study was to test the psychometric properties of the Chinese version of the BRIAN (C-BRIAN) in college students with clinically significant depressive symptoms. We hypothesized that the C-BRIAN would have good construct and convergent validity, as well as good internal consistency.

## Method

### Study design

The data were extracted from a pre-existing project, “Promoting Mental Health by Physical Activity in Adolescents”, which is a prospective naturalistic observational study investigating how physical activity impacts daily mood using an ecological momentary assessment and Polar Unite in college students. A video introducing our project was pre-recorded and the teachers showed it to the college students during class time. Students then gave their informed consent and filled out the questionnaire on their mobile phones using “REDCAP”, which is an online data collecting and administration platform. The assessment items included in the questionnaire generally tapped on depression, anxiety, biological rhythms, suicidal and self-harm behaviors, and ideation (detailed in “Participants” and “Assessments” below). Based on to the baseline scores from the Center for Epidemiological Studies Depression Scale (CES-D), we divided the college participants into two groups: 1) participants with depressive symptoms and 2) healthy control. Construct and convergent validity as well as the internal consistency were all assessed.

All procedures contributing to this work comply with the ethical standards of the relevant national and institutional committees on human experimentation and with the Helsinki Declaration of 1975, as revised in 2008. All procedures involving human subjects were approved by the Institutional Review Board of The Affiliated Brain Hospital of Guangzhou Medical University (reference 2021-089). Written informed consent from all subjects was obtained using “REDCAP” and the participation in this study was voluntary.

### Participants

All participants aged 18–24 years included in current validation analysis studied at the Sport Training College of Guangzhou Sport University. They were assessed by the Center for Epidemiological Studies Depression Scale (CES-D), and those that scored 16 or greater were considered as the depressive group (23), with the rest treated as the healthy control group. The exclusion criteria were as follows: 1) student reporting psychoactive substance use history or diagnosed with mental disorders excluding mood disorders, generalized anxiety disorder, insomnia, and advanced/delayed sleep phase syndrome; 2) severe physical diseases including but not limited to organic brain diseases; 3) history of head trauma; 4) current hypnotic sedative drug use as indicated by selecting the seventh item of the Pittsburgh Sleep Quality Index (PSQI); or 5) life events in the past month that could alter sleep habits (e.g., preparing for a competition; shift work; in hospital, etc.).

A total of 710 students were invited to participate in the present study, and we collected 531 participants’ informed consent. After checking for the completeness of the data, only 387 participants had completed the online questionnaire fully, with eight students currently using hypnotic or sedative drugs and one student diagnosed with a mental disorder excluded from the data analysis. Therefore, a total of nine participants were excluded from the current study. Only two participants reported bipolar disorder diagnoses and they were not excluded. Taken altogether, 292 individuals categorized into the depressive symptoms group, and 86 individuals were treated as the healthy control group.

### Assessments

The BRIAN is a self-report questionnaire comprised of 21 items which tap on five domains. The scores of items 1–18 make up the total score, while items 19–21, the fifth domain, measure chronotype (18). Meanwhile, the items focusing on the first four domains assess sleep, general activity, social activity, eating patterns, and whether the individual has experienced disturbances in these rhythms and how frequently over the past 15 days, rating each item as 1(not at all), 2 (seldom), 3 (sometimes), and 4 (often). The total score can range from 18 to 72 with higher BRIAN scores indicating more severe circadian rhythm disturbance.

To establish the validity and reliability for the Chinese version of the BRIAN, one of the Chinese researchers in the current research team formed by experienced and practicing psychiatrists first translated the English version into Chinese. Back-translation was performed by the other Chinese researcher in the team who was not already familiar with the BRIAN. Three experienced clinical specialists majoring in mood disorders gave us further suggestions for modifications to make the measure more suitable for the Chinese young college population. The permission to use BRIAN in the present study was already sought from the original author, Prof. Flávio Kapczinski. The original version of BRIAN was published in a peer-reviewed journal (18). Similar to another study translating BRIAN into Arabic (24), the present study was approved to use and translate this scale for research purposes as long as there was the citation of the original publication to give the credit to the original authors. The C-BRIAN scale used in this current study was a translated scale by our research team targeting college students (aged between 18 and 24 years old) who had subclinical depressive symptoms in the general public; while the version used by He et al. (22) was translated and validated in clinical patients with MDD aged around 40 years old. Given that the age range and clinical profile of the target group in this current study was very different from previous validation study (22), it is important to translate the C-BRIAN specifically for the participants in the current study. To evaluate the severity of depressive symptoms, we used the CES-D, which has been designed to assess depressive symptomatology in the general population (23). The cutoff score for the CES-D is 16, with previous research studies showing that 70% of patients but only 21% from the general population scored at or higher than the cutoff score (23).

The MEQ is used widely to measure human circadian rhythms (17). While the reduced version contains only five questions compared to the 19 questions in the original version, the short version (rMEQ) has been shown to be more suitable for data collecting from broader samples of subjects while still highly correlating with the original MEQ (25). As such, the reduced version was chosen to verify the convergent validity of C-BRIAN in the current study. The Pittsburgh Sleep Quality Index (PSQI) is the most commonly used tool for assessing sleep quality, and has been shown to have strong reliability and validity (16, 26). Finally, seven items of the CES-D measuring the somatic and retarded activity factors were used (i.e., items 1, 2, 5, 7, 11, 13, and 20), which was supported by previous studies (23, 27). Both the PSQI and some items from the CES-D were applied to further verify convergent validity.

### Statistical analyses

SPSS 25.0 for Windows was used to perform statistical analyses in the present study. A p-value was set at.05, and any values less than this was considered to be a statistically significant difference. Demographic and baseline data from both the depressive symptoms and control groups were compared using chi-square and t-tests. To evaluate the construct validity of the C-BRIAN, explorative factor analysis (EFA) was applied. Since the factor properties of BRIAN in Chinese college student population with depressive symptoms was not investigated before, an explorative factor analysis (EFA) instead of a confirmative factor analysis was applied to test the C-BRIAN construct with the current sample. First, the Kaiser-Meyer-Olkin (KMO) measure of sampling adequacy and Bartlett’s test were used to determine whether high correlations existed between items 1–18. If KMO values were close to 1 and had a p-value on the Bartlett’s test of less than .05, the data were deemed suitable for factor analysis. The principal components method was then used, and we considered both the scree plot and eigenvalues to select factor numbers. Finally, we observed whether the extracted factors could be explained by the relevant items and whether they corresponded to the original design. The PSQI and rMEQ were used to assess convergent validity. The scores from the seven extracted items of the CES-D were also applied to assess convergent validity. We added up the scores for the sleep domain of the C-BRIAN (i.e., items 1 to 5) and bivariate correlations were applied. The values for the Pearson’s correlation were set as follow: no correlation (0–0.3), weak (0.3–0.5), moderate (0.5–0.8), strong (0.8–1). The process was the same for the somatic/activity domain (i.e., items 6 to 18) and the chronotype domain (i.e., items 19 to 21); items 19 and 21 used reverse scoring so that scores were consistent with the rMEQ. The internal consistency for each factor analysis subscale was assessed by calculating Cronbach’s alpha coefficient.

## Results

### Comparison about demographic and characteristics between two groups

As **Table 1** shows, there were no notable differences between the depression and control groups with regards to age (20.8 ± 1.3 vs. 20.7 ± 1.3, *p*=0.665), percentage of males (59.9% vs. 60.5%, *p*=0.929), BMI (21.9 ± 3.2 vs. 22.0 ± 2.6, *p*=0.654), and rMEQ (14.2 ± 3.3 vs. 14.8 ± 2.5, *p*=0.099). The depression group reported significantly higher CES-D scores (22.2 ± 5.6 vs. 12.1 ± 2.0, *p*<0.001) and mean BRIAN scores (30.0 ± 10.5 vs. 27.0 ± 6.7, *p*< 0.001). The high prevalence of depressive symptoms (41.1%) among college students found in the current study (conducted during the pandemic) was similar to what was found in other studies in Chinese after the pandemic (48.4%) (e.g., 4). Moreover, all participants, who were assessed during the pandemic in the present study, were athletes studying at the Sport Training College of Guangzhou Sport University. During the pandemic, mainland Chinese government had strict lockdown law and regulations in Guangzhou which prohibited majority of people from going out of home for school, leisure and sports activities, which might particularly worsen the mental health and depressiveness in the participants who were athletes at the time of assessment. In summary, it was the pandemic and lockdown regulations that might explain why a high proportion of participants was categorized into the depressive symptoms group in the present study. Meanwhile, the PSQI scores showed that those in the control group had significantly better sleep quality than those with depressive symptoms (4.0 ± 2.2 vs. 5.4 ± 2.8, *p*=0.007).

**Table 1:** Demographic and characteristics of the two groups.

	Depression (*n*=292) N(%) or Mean(SD)	Control (*n*=86) N(%) or Mean(SD)	*p*-value
Age(years)	20.8(1.3)	20.7(1.3)	0.665
Male(%)	175(59.9)	52(60.5)	0.929
BMI(kg/m2)	21.9(3.2)	22.0(2.6)	0.654
CES-D(points)	22.2(5.6)	12.1(2.0)	<0.001**
PSQI(points)	5.4(2.8)	4.0(2.2)	0.007*
rMEQ(points)	14.2(3.3)	14.8(2.5)	0.099
C-BRIAN(points)	30.0(10.5)	27.0(6.7)	<0.001**

C-BRIAN, Chinese Version of the Biological Rhythms Interview of Assessment in Neuropsychiatry; BMI, Body Mass Index; CES-D, Center for Epidemiological Studies Depression scale; PSQI, Pittsburgh Sleep Quality Index; rMEQ, reduced Morningness-Eveningness Questionnaire.

*≤0.05, **≤0.001.

### Construct validity of the C-BRIAN

**Figure 1:**
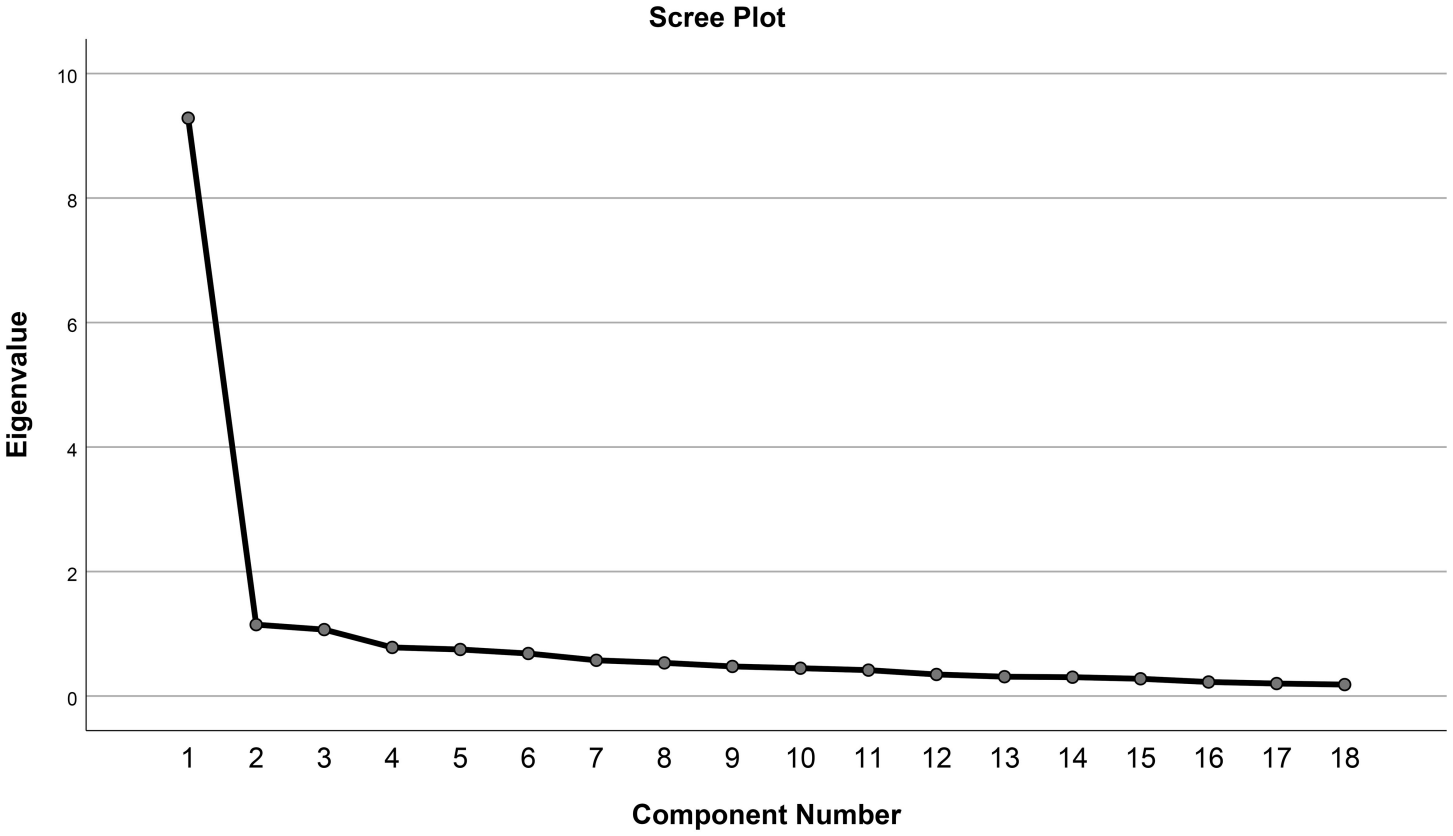
Scree plot.

The KMO value was 0.944 which is very close to 1, and Bartlett’s test showed that the p-value was less than 0.001, indicating that the data was appropriate for factor analysis. The scree plot shown in **Figure 1** suggested two to three factors should be extracted, while three components’ eigenvalues were greater than 1. As a result, three factors were extracted as shown in **Table 2**, explaining 63.9% of the total variance. The first factor comprised 10 items (i.e., items 6, 7, 8, 9, 10, 11, 12, 13, 14, and 18), and was primarily about physical and social activities. The second factor comprised three items (i.e., items 15, 16, and 17) which related to eating patterns. The third factor comprised six items (i.e., items 1, 2, 3, 4, 5, and 6) which assessed sleep disturbance. Item 6 was included in both factors one and three, and its factor three loading (0.593) was somewhat higher than that in factor one (0.579). In addition, the change of total explained variance was minimal when four factors were considered (68.2%) and therefore, three-instead of four-factor structure was proposed according to the scree plot and EFA results. A confirmative factor analysis should be applied in future studies to test the 3-factor structure.

**Table 2:** Factor loadings and reliability of the C-BRIAN factors.

Item number	Factor		
	Activities/Social scale	Eating Patterns scale	Sleep scale
BRIAN8 (maintaining physical activity)	0.759		
BRIAN7 (completing household activities)	0.757		
BRIAN 10 (maintaining sexual activity/libido)	0.727		
BRIAN 9 (maintaining scheduled activities)	0.648		
BRIAN 11 (communicating with others)	0.647		
BRIAN 13 (synchronizing with others)	0.639		
BRIAN 12 (overusing electronic devices)	0.590		
BRIAN 18 (using stimulants with moderation)	0.573		
BRIAN 14 (giving attention to others)	0.553		
BRIAN 16 (avoiding skipping meals)		0.838	
BRIAN 15 (keeping meal times)		0.822	
BRIAN 17 (eating regular amounts)		0.785	
BRIAN 4 (feeling rested)			0.731
BRIAN 1 (falling asleep)			0.711
BRIAN 5 (switching off)			0.660
BRIAN 6 (completing work activities)	0.579		0.593
BRIAN 3 (getting out of bed)			0.530
BRIAN 2 (wakingup)			0.511
Cronbach’s α	0.911	0.893	0.909

Factors were extracted using principal component analysis and rotated by the varimax method. C-BRIAN, Chinese Version of the Biological Rhythm Interview for Assessment in Neuropsychiatry.

### Convergent validity of the C-BRIAN

**Figure 2:**
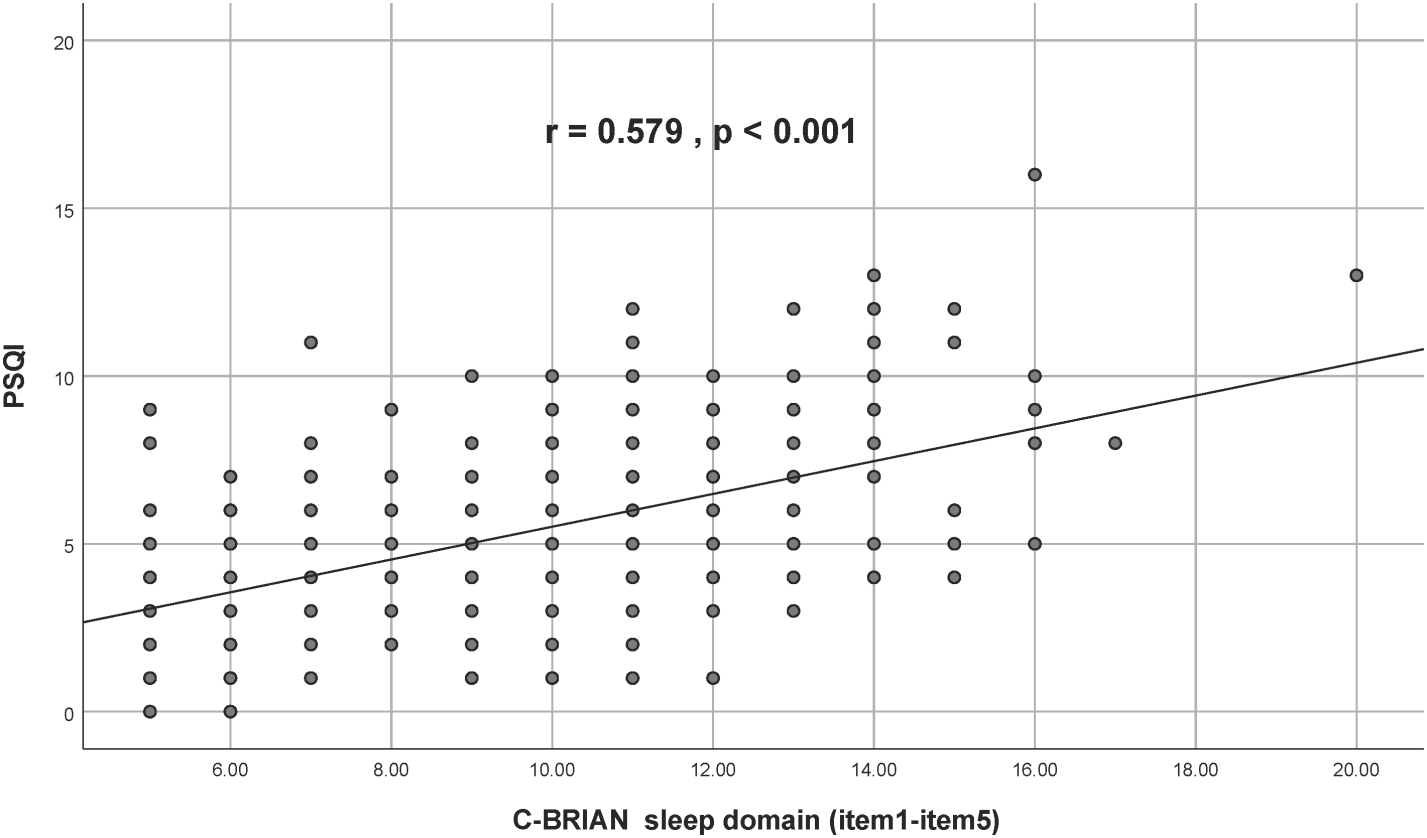
Association Between Sleep Domain of C-BRIAN and PSQI Global Score. There was a significantly moderate strength correlation between the sleep domain of the C-BRIAN and the PSQI. C-BRIAN, Chinese Version of the Biological Rhythms Interview of Assessment in Neuropsychiatry; PSQI, Pittsburgh Sleep Quality Index.

**Figure 3:**
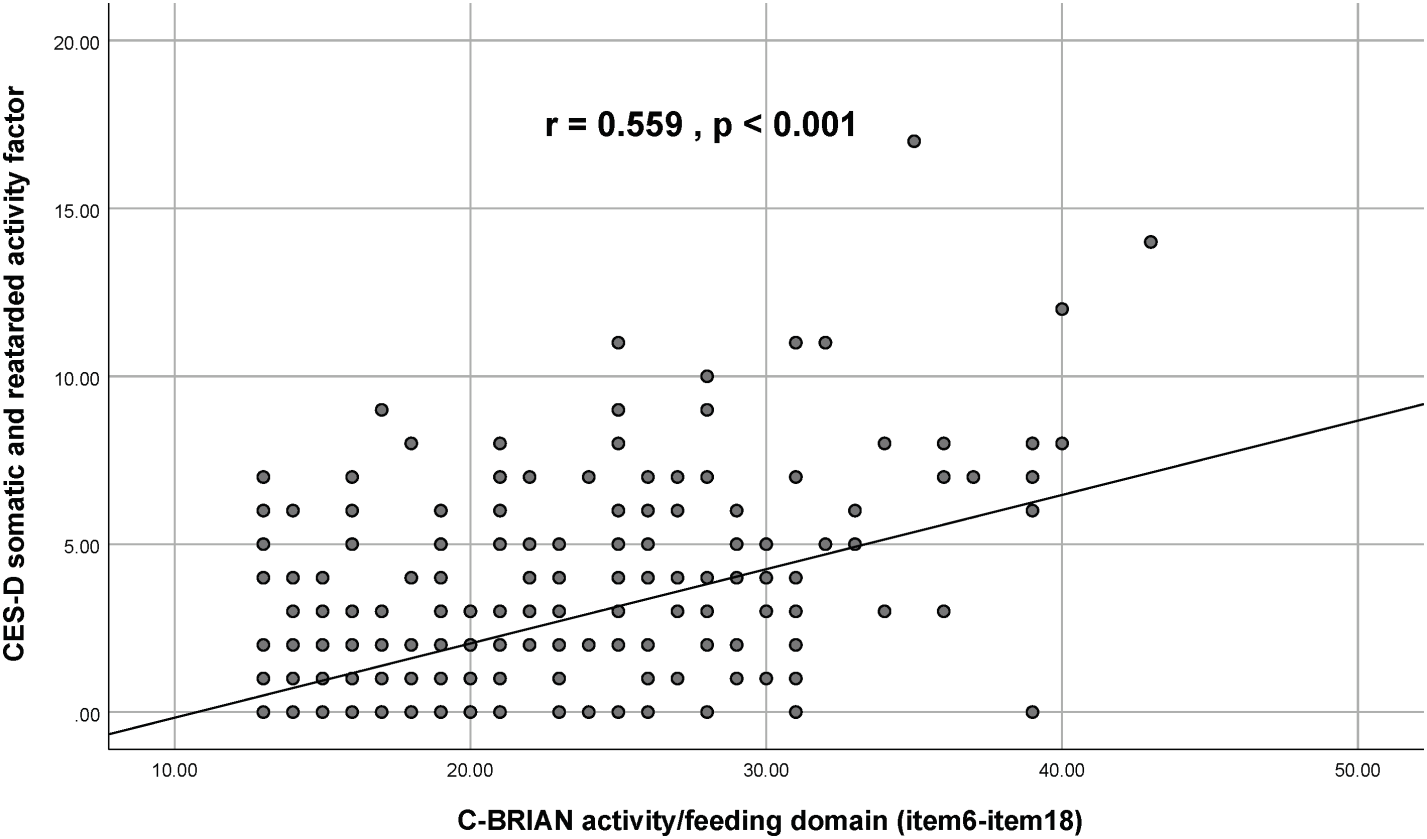
Association Between Activity/Eating Patterns Domain of the C-BRIAN and Somatic and Retarded Activity Factor of the CES-D. Somatic and retarded activity factor of the CES-D comprised items 1,2,5,7,11,13, and 20 from the CES-D. Results indicated that there was a significantly moderate strength correlation between the two. CES-D, Center for Epidemiological Studies Depression Scale.

**Figure 4:**
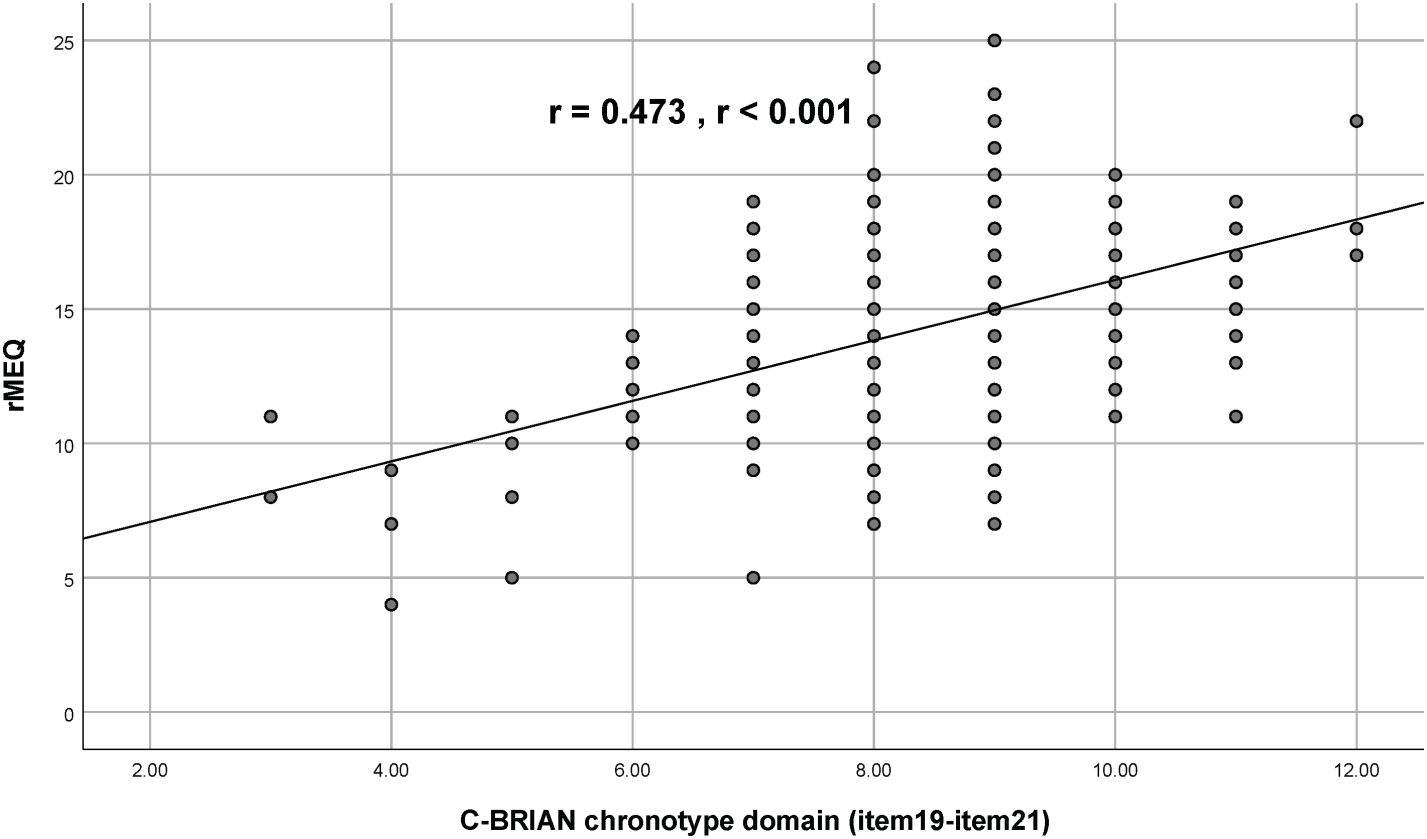
Association Between Chronotype Domain of the C-BRIAN and the rMEQ. A weak strength correlation was observed. RMEQ, Reduced Morningness-Eveningness Questionnaire.

Visual representation of the relationship between the C-BRIAN and the other measures can be seen in **Figures 2**–**4**. The Pearson’s correlation between sleep domain scores (i.e., items 1 through 5) and the PSQI was 0.579, and its p-value was less than 0.001, showing a statistically significant and moderate strength of correlation (**Figure 2**). This result was similar to the relationship between the physical and social activity scale and the eating patterns scales of the C-BRIAN (i.e., items 6 to 18) as well as the somatic and retarded activity factor scales of the CES-D (i.e., item 1, 2, 5, 7, 11, 13, and 20) (r=0.559, *p*<0.001, **Figure 3**). As for chronotype domain and rMEQ, a weak strength correlation was revealed (r=0.473, *p*<0.001, **Figure 4**). Our results indicated that the C-BRIAN had good convergent validity.

### Internal consistency of the C-BRIAN

With regard to the overall C-BRIAN (all 18 items), Cronbach’s alpha coefficient was 0.94 indicating a very good internal consistency. The Cronbach’s alpha coefficients of each subscale extracted by factor analysis were 0.91, 0.89, and 0.91, corresponding to the first factor, second factor, and third factor, respectively (**Table 2**). Regarding the Split-Half reliability test for C-BRIAN and Cronbach’s α values for each item, we split all items into two sections: odd number section and even number section. Then, we calculated the correlations between items on these two sections. For the odd number section, the Cronbach’s α coefficient was 0.91. For the even number section, Cronbach’s α coefficient was 0.86. Importantly, Guttman split-half coefficient was 0.94 which indicated a very good internal consistency. In comparison, using original BRIAN scoring scheme with 5 domains, the Cronbach’s alpha coefficients were 0.79 (sleep domain: item 1 to item 5), 0.87 (activity domain: item 6 to item 10), 0.86 (social domain: item 11 to item 14), 0.85 (eating pattern domain: item 15 to item 18) and 0.65 (chronotype domain: item 19 to item 21) respectively. The current study with newly proposed 3-factor structure showed a better internal consistency (0.89–0.91) for C-BRIAN among the current sample with college students.

## Discussion

Given that young adults, specifically college students have high prevalence of depressive symptoms, it is essential to develop a reliable and valid measurement tool for biological rhythms which can serve as an early detection of depression for timely and efficient treatment in Chinese community who are non-clinical individuals. The present findings have a number of implications. Most importantly, it provided significant support to the psychometric property and appropriateness of C-BRIAN in assessing biological rhythms in Chinese college students. Moreover, the present results showed that even though depressive symptoms may exist in young adults (possibly without a diagnosis), the biological rhythms of students in the depressive symptom group showed higher levels of disturbance in terms of biological rhythms than those of students in the control group, reflected by the total C-BRIAN scores. Future studies should investigate the role of biological rhythm in the development of mood symptoms or disorders, thereby enhancing community mental health.

In the present study with Chinese young adults from the community, the viability and reliability of the C-BRIAN in a non-clinical setting is established. Specifically, three factors from the C-BRIAN were extracted which are similar to those factors extracted from the original and Japanese versions of the BRIAN (18, 21). However, compared to these other versions, the specific factor compositions in the C-BRIAN were different. In the present study, the items regarding activity and social domains comprised an independent factor. The probable cause of different composition of the factors among previous studies might be that the samples investigated were different, and that they had diverse characteristics. For instance, attenuated amplitude of daily physical activity was found in depressed individuals, and sedentary behavior such as prolonged TV viewing or computer/internet use increased one’s risk of depression, and that all of these can also be acts of social exclusion (28, 29). Physical activity probably increased one’s opportunity of exposure to social situations (30), while maintaining regular social rhythm required necessary physical activities. This bidirectional relationship may become more remarkable in a depressed population. However, due to memory bias or additional effects because of other variables (31), a more ecological assessment method should be applied for further investigations. To extend the findings from previous validation studies with clinical patients (e.g., 22), the present study provides support to the validation of C-BRIAN in the general public with depressive symptoms which has important implications. In addition, previous studies used global BRIAN scores to test convergent validity while the psychometric properties of the scores of each subscale were examined in the present study. Specifically, all correlations were found statistically significant, suggesting that the C-BRIAN has good convergent validity. There are both advantages and disadvantages of investigating the narrow age range in the current study. For instance, the current findings might be specifically applicable to university students or young adults who are vulnerable to depression and mood problems. Also, all participants came from the same institution, which could introduce bias into the results. Therefore, it is important to consider these when adopting C-BRIAN in other age groups such as older adults.

With regard to the relationship between biological rhythms and depressive symptoms, it is noteworthy that the current study found more reports of poor sleep quality in the depressive symptom group. These results are consistent with previous findings (e.g. (15), For instance, a meta-analysis on depression suggesting that the risk of first onset of depressive disorder in adolescence or early adulthood is 1.62 times higher in people who have experienced sleep disturbances compared to those who have never experienced sleep disorders (10). As for chronotype, there was a slight tendency towards delayed sleep in the depressive symptom group, although both groups did not show obvious chronotype in the present study which was also consistent with previous study. For instance, a neuroimaging study found that a delayed chronotype enhanced amygdala reactivity and reduced fronto-limbic functional connectivity, while a longitudinal study indicated that an eveningness chronotype was associated with more severe depressive and anxiety symptoms (32, 33). Not only that the present findings are consistent with prior findings, but the present results also suggested that an early intervention should be implemented to reset the rest-activity pattern in those experiencing delayed sleep patterns to prevent this trend running its course and thereby lowering risks of developing depression. Indeed, a randomized clinical trial has already found that doing this has a positive impact (34). Moreover, Cruz-Sanabria et al. (35) showed that exogenous melatonin on sleep and circadian parameters had a significant effect on bipolar disorder (BD) and delayed sleep-wake phase disorder patients (DSWPD). These findings signify the importance of targeting circadian rhythms in people with depression or mood disorders.

The strengths of the present study are that we incorporated a relatively large sample size in non-clinical setting, and that our results reach a very good level of validity as recommended by COSMIN’s criteria (36). There are, however, still some limitations. First, our sample population was limited to college student athletes and the age range was relatively narrow. Future research should use broader samples with wider demographic variety to further examine the properties of the C-BRIAN in different adult age groups. Second, the assessment of depression relied on a self-report questionnaire which lacked an objective assessment. Future research should be performed in a clinical setting. Third, we didn’t exclude participants with diagnoses of bipolar disorder, which can result in different manifestations of rhythm disturbance from the general depression state or depressive disorders. However, only two participants reported having bipolar disorder, so this likely had little influence on our findings. These two participants with bipolar disorder diagnoses showed significant depressive symptoms assessed by CES-D and hence they were included to the current study because the main purpose of our study was to evaluate the validity and reliability of C-BRIAN in a non-clinical setting which may include individuals with different mood and psychological disorders such as bipolar disorder. Although patients in manic/hypomanic phase might present different manifestation in circadian rhythm, the current study with only 2 bipolar patients could not investigate whether a unique circadian rhythm disturbance manifestation existed in bipolar depression. Last but not least, this study was conducted during pandemic and the original plan included a research design for test-retest reliability. However, due to the frequent lockdowns and strict quarantine measures in Mainland China during when this study was conducted, the retest of C-BRIAN and other measures was not successfully administered within the expected timeframe and the participants might also have difficulty in the chronological adjustment during the lockdown. Future studies should also consider conducting the test-rest reliability for C-BRIAN and other measures adopted in this study in the post-pandemic time.

## Conclusion

The psychometric properties of the C-BRIAN were examined using a college population with or without depressive symptoms. Findings of the present study showed that C-BRIAN has a very good internal consistency as well as good construct and convergent validity. Our study indicates that the C-BRIAN can serve as a useful and reliable instrument in evaluating the severity of biological disturbances in depressed young adults in the community, while its application in clinical settings and a wider age demographic requires further exploration. Nonetheless, the establishment of a reliable and valid measurement tool for biological rhythms, specifically C-BRIAN would help detect depressive symptoms for timely prevention and intervention in the community which have important clinical implications benefiting both subclinical and clinical individuals.

## Data availability statement

The raw data supporting the conclusions of this article will be made available by the authors, without undue reservation.

## Ethics statement

The studies involving humans were approved by Institutional Review Board of The Affiliated Brain Hospital of Guangzhou Medical University (reference 2021-089). The studies were conducted in accordance with the local legislation and institutional requirements. The participants provided their written informed consent to participate in this study.

## Author contributions

HH: Methodology, Writing – original draft. XT: Data curation, Methodology, Writing – review & editing. BY-HL: Writing – review & editing. WL: Methodology, Writing – review & editing. XL: Formal analysis, Writing – review & editing. SH: Conceptualization, Writing – review & editing. XX: Writing – review & editing. RZ: Formal analysis, Writing – review & editing. RW: Formal analysis, Writing – review & editing. DL: Conceptualization, Writing – review & editing. YG: Conceptualization, Writing – review & editing. NC: Methodology, Writing – review & editing. SW: Methodology, Writing – review & editing. GX: Methodology, Writing – review & editing. KL: Conceptualization, Methodology, Writing – review & editing.
